# Evaluation of Styrene-Divinylbenzene Beads as a Support to Immobilize Lipases

**DOI:** 10.3390/molecules19067629

**Published:** 2014-06-10

**Authors:** Cristina Garcia-Galan, Oveimar Barbosa, Karel Hernandez, Jose C. S. dos Santos, Rafael C. Rodrigues, Roberto Fernandez-Lafuente

**Affiliations:** 1 Departamento de Biocatalisis, ICP-CSIC, Campus UAM-CSIC, Cantoblanco, 28049 Madrid, Spain; E-Mails: c.garcia@icp.csic.es (C.G.-G.); jscleiton@gmail.com (J.C.S.D.S.); 2 Escuela de Química, Grupo de investigación en Bioquímica y Microbiología (GIBIM), Edificio Camilo Torres 210, Universidad Industrial de Santander, Bucaramanga 680001, Colombia; E-Mail: oveimar@gmail.com; 3 Biotransformation and Bioactive Molecules Group, Instituto de Química Avanzada de Cataluña-CSIC Jordi Girona 18-26, 08034 Barcelona, Spain; E-Mail: khs27883@gmail.com; 4 Departamento de Engenharia Química, Universidade Federal do Ceará, Campus do Pici, CEP 60455-760 Fortaleza, CE, Brazil; 5 Biotechnology, Bioprocess and Biocatalysis Group, Institute of Food Science and Technology, Federal University of Rio Grande do Sul, Av. Bento Gonçalves, 9500, P.O. Box 15090, ZC 91501-970, Porto Alegre, RS, Brazil; E-Mail: rafaelcrodrigues@ufrgs.br

**Keywords:** lipase immobilization, modulation of lipase activity, interfacial activation, styrene divinylbencene matrix

## Abstract

A commercial and very hydrophobic styrene-divinylbenzene matrix, MCI GEL^®^ CHP20P, has been compared to octyl-Sepharose^®^ beads as support to immobilize three different enzymes: lipases from *Thermomyces lanuginosus* (TLL) and from *Rhizomucor miehie* (RML) and Lecitase^®^ Ultra, a commercial artificial phospholipase. The immobilization mechanism on both supports was similar: interfacial activation of the enzymes *versus* the hydrophobic surface of the supports. Immobilization rate and loading capacity is much higher using MCI GEL^®^ CHP20P compared to octyl-Sepharose^®^ (87.2 mg protein/g of support using TLL, 310 mg/g using RML and 180 mg/g using Lecitase^®^ Ultra). The thermal stability of all new preparations is much lower than that of the standard octyl-Sepharose^®^ immobilized preparations, while the opposite occurs when the inactivations were performed in the presence of organic co-solvents. Regarding the hydrolytic activities, the results were strongly dependent on the substrate and pH of measurement. Octyl-Sepharose^®^ immobilized enzymes were more active *versus* p-NPB than the enzymes immobilized on MCI GEL^®^ CHP20P, while RML became 700-fold less active *versus* methyl phenylacetate. Thus, the immobilization of a lipase on this matrix needs to be empirically evaluated, since it may present very positive effects in some cases while in other cases it may have very negative ones.

## 1. Introduction

Lipases are the most used enzymes in biocatalysis, both at the industrial and academic levels [1,2,3,4,5]. This is because lipases are quite robust under a wide range of conditions and reaction media (including organic solvents, ionic liquids or supercritical fluids) [6,7], have a broad specificity accepting very different substrates (while presenting in some instances a high regio and enantio selectivity and specificity) [8,9], and can catalyze many interesting reactions (hydrolysis of esters, esterification, transesterification) [10,11,12,13] and even some promiscuous reactions (perhydrolysis, carbon-carbon bonds formation) [14,15,16].

Even with these good initial prospects, the properties of lipases need to be improved, as for many other enzymes, to be used as industrial biocatalysts. Enzyme immobilization, if properly utilized, may improve many features, from stability to activity and specificity [17,18,19,20,21,22]. In some cases, the one-step immobilization-purification of the enzyme may increase the impact of the immobilization step, because in industry, pure enzymes are hardly utilized and in some cases, this may generate some problems [23].

Lipases present a peculiar catalytic mechanism, called interfacial activation [24,25,26]. In most cases, their active center is isolated from the medium by a polypeptide chain called lid (closed form) [27]. In some cases, the lid is so small that it cannot seclude the active center from the medium, as it is the case of the lipase B from *Candida antarctica* (CALB) [28]. In most cases, the lid is able to fully isolate the active center, and even in some cases a double lid has been described [29]. The internal face of the lid is hydrophobic and is interacting with the hydrophobic areas around the active center. The lid can move, exposing the active center as well as this large hydrophobic pocket, in an equilibrium shifted towards the closed form [24,25,26]. In the presence of drops of the natural substrate (oils), the hydrophobic pocket of the open form is stabilized after enzyme adsorption and the enzyme becomes adsorbed and oriented on the substrate. It has been shown that lipases are adsorbed on any hydrophobic surface, including hydrophobic proteins [30], open forms of other lipase molecules [31,32], or hydrophobic supports [33].

The immobilization of lipases on hydrophobic supports has been reported as a very efficient way to immobilize, purify and hyperactivate lipases, allowing retention of the open form of the enzyme without any external interface [33]. This way, the lipases immobilized following this strategy will have the open structure stabilized by interactions with the support, involving the hydrophobic area of the lid and the hydrophobic areas surrounding the active center [33]. It has also been shown that the nature (internal morphology, hydrophobicity of the surface, *etc.*) of the support may greatly affect the final properties of the immobilized enzyme (activity, stability, even selectivity and specificity) [34,35].

Recently, a commercial matrix composed of styrene divinylbenzene (MCI GEL^®^ CHP20P) allowed great improvement of some properties of the lipase B from *Candida antarctica* (CALB) [36], perhaps the most popular enzyme in biocatalysis [37]. Enzyme activity *versus* many substrates could be improved when comparing this immobilized catalyst to the commercial one or even other home-made preparation based on similar immobilization mechanism. Stability in co-solvents was improved, while thermo-stability was lowered using this very hydrophobic matrix [36]. The loading capacity of the support using CALB was also very high. These immobilized biocatalysts were used in other reactions exhibiting in many cases some advantages: in some instances higher activity was found, in other instances a better operational stability was detected [38,39,40,41]. Even after this preliminary success using CALB, few examples on the use of this support to immobilize other enzyme may be found in the literature. The lipase from *Thermomyces lanuginosus* has been utilized in esterification reactions after immobilization on this support [42], but the biocatalyst properties (activity, stability, loading capacity), have not been analyzed. In another paper, the purification/immobilization of the lipase from *Staphylococcus warneri* EX17 on this support was shown [43].

Now, in this new paper, we intended to present the prospects of this new support to immobilize the lipases from *Thermomyces lanuginosus* (TLL) [44] and the lipase from *Rhizomucor miehei* (RML) [45,46], very likely some of the most popular lipases after CALB. We have also included Lecitase^®^ Ultra (LU) in these studies, a commercial chimeric phospholipase built from the gen of the lipase from *Thermomyces lanuginosus* (to obtain good stability) and that of the phospholipase from *Fusarium oxysporum* (to get the phospholipase activity) [47]. As reference, we will use octyl-Sepharose^®^ beads, a support reported as very useful to immobilize lipases via this strategy [48]. The loading capacity of the support and activity *versus* several substrates, as well as the stability in diverse conditions was analyzed for all biocatalysts to advance on the prospects of this matrix as a general support to immobilize lipases.

## 2. Results and Discussion

### 2.1. Immobilization Courses of the Different Lipases on Both Supports

**Figure 1:**
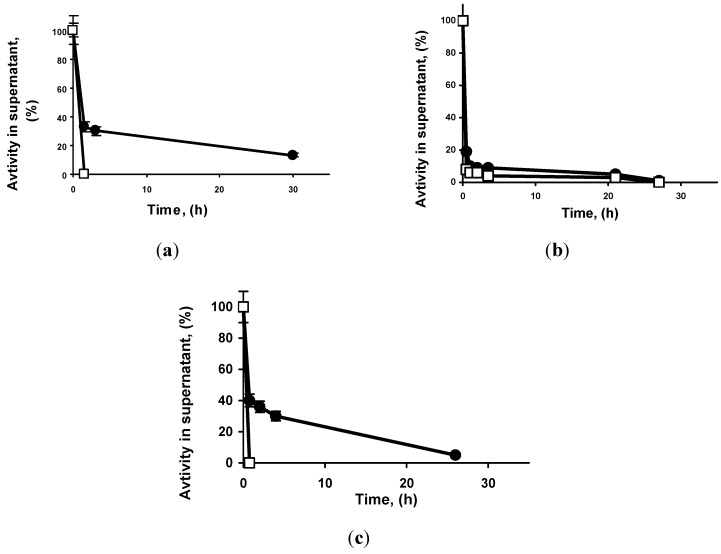
Immobilization courses of different enzymes on octyl-Sepharose^®^ and MCI GEL^®^ CHP20P beads. The activity of the supernatants is shown (free enzyme remained fully active under immobilization conditions). Experiments were carried out as described in methods, using a support/total volume ratio of 0.1 and a loading of 3 mg of protein/g of support. A: TLL, B: RML, C: LU. Circles: octyl-Sepharose^®^, Squares: MCI GEL^®^ CHP20P. The results are the mean of 3 independent experiments and the experimental error was never over 10%.

Figure 1 shows the immobilization courses of the three enzymes on octyl-Sepharose^®^ and MCI GEL^®^ CHP20P beads. The immobilization was always faster using MCI GEL^®^ CHP20P, in some cases differences are clearer (TLL), in other cases the differences are lower (RML). In fact, using TLL the immobilization yield using octyl support is around 80% after 24 h, while using the new support the yield is 100% just after some few minutes of contact. This should be related to the more hydrophobic nature of this support, the support must make a competition with the other lipase molecules, that can also form bimolecular aggregates via interaction with two open forms of the lipase [31,49] to immobilize the enzymes. In Table 1 the values of the loading capacity of the new support are given, considering a maximum immobilization time of 24 h. The loading using the new supports (87.2 mg protein/g of support using TLL, 310 mg/g using RML and 180 mg/g using Lecitase^®^ Ultra) is nearly thirty-fold higher than that observed using octyl-Sepharose^®^.

**Table 1:** Loading observed using the different enzymes on octyl-Sepharose^®^ and MCI GEL^®^ CHP20P.

Lipase/support	Octyl-Sepharose^®^	MCI GEL^®^ CHP20P
**RML**	11 ± 2	310 ± 20
**TLL**	20 ± 2	90 ± 8
**LU**	30 ± 3	180 ± 15

The loadings are given in mg of protein per gram of wet support. Experiments were performed as described in Experimental section. The results are the mean of three independent experiments and the experimental error was never over 10%.

### 2.2. Effect of pH on Immobilized Enzyme Activities Versus pNPB

All studied lipases are hyperactivated after immobilization on octyl-Sepharose^®^, RML multiplied the activity by 3.8, TLL by 1.65 and Lecitase^®^ Ultra by 2 (results not shown). The increase in lipase activity after immobilization on octyl-Sepharose^®^ has been previously reported and it has been explained by the stabilization of the open form of the lipase [48]. The observed increment in activity after the immobilization depends on the loading of the immobilized biocatalyst (because diffusion problems may decrease the observed activity after immobilization) and the concentration of the free lipase in the activity measurements (because this determines the dimerization of the lipase and can cause an alteration of the detected activity ), *etc.* [31,49].

Table 2 shows the activity data for the 3 enzymes immobilized on both supports in a pH range from 5 to 10, using a loading of only 3 mg/g of support. It should be considered that the free enzymes have a tendency to produce aggregates, therefore they may generate results, which could be very hard to interpret [32,50]. RML activity is around 20-fold lower when immobilized on MCI GEL^®^ CHP20P than when immobilized on octyl-Sepharose^®^. However, the pH/activity curve is rather similar. TLL activity is also lower using MCI GEL^®^ CHP20P, but the differences are shorter. The optimal pH value is similar for both biocatalysts. Nevertheless, the new biocatalyst seems to retain more activity at alkaline pH values. The results were even more negative using Lecitase^®^, the drop in activity was of more than 100 fold factor. Moreover, the optimal pH was 6 for MCI GEL^®^ CHP20P-Lecitase^®^, and 10 for the octyl preparation.

**Table 2:** Specific activities of the different biocatalyst *versus* pNPB under different conditions.

Biocatalyst	pH					
	5	6	7	8	9	10
**TLL-OS**	26	33	34	35	55	38
**TLL-MCI**	1.38	1.39	1.41	3.72	4.66	4.44
**RML-OS **	9.7	11.25	12.1	10.2	6.4	1.3
**RML-MCI**	0.45	0.45	0.50	0.47	0.33	0.02
**LU-OS**	10.5	13	11.5	16.5	16.8	28
**LU-MCI**	0.16	0.50	0.42	0.37	0.36	0.45

For a better comparison, loadings of 3 mg of protein/g of wet support were used in all cases. OS: Octyl Sepharose^®^; MCI: MCI GEL^®^ CHP20P. Specific activity is defined as μmol of pNP released per minute and mg of immobilized enzyme. Experiments were performed as described in methods section. The results are the mean of three independent experiments and the experimental error was never over 7%.

It is not easy to explain this difference in activity *versus* pNPB using the new support and octyl-Sepharose^®^, considering that in both cases a similar immobilization mechanism is expected, involving interfacial activation *versus* the hydrophobic support [48]. It is also curious that the pH/activity profile may have some clear differences. The main difference between both supports is the internal morphology (trunks for agarose, tunnels for MCI GEL^®^ CHP20P). In the MCI support, adsorption takes place directly on the matrix and not in a hydrophobic layer over a hydrophilic matrix, which makes MCI much more hydrophobic than octyl-Sepharose^®^. Moreover, in the MCI support the enzyme is interacting with the surface, not with acyl groups that can place some distance between enzyme and support wall. Thus, one possible explanation is the occurrence of some partition effect of the substrate, which is an aqueous solution, from the hydrophobic matrix of MCI, leading to lower activity on this support. Additionally, it has been previously reported that the differences on the immobilization support may be enough to explain differences on lipases immobilized via interfacial activation on hydrophobic supports [35]. This way, it is expected that some conformational changes induced by the very hydrophobic surface will lead to different activities [20,51].

### 2.3. Thermal Stability of MCI GEL^®^ CHP20P and octyl-Sepharose^®^ Lipase Preparations

Table 3 shows the negative effect of the immobilization on MCI GEL^®^ CHP20P of all the enzymes on their thermal stability. In general, differences in half-lives were shorter at pH 9 and much higher at pH 5 and 7. The MCI preparations fully lost the activity under conditions where the octyl-Sepharose^®^ preparations retained a high percentage of the initial activity; this was more dramatic using TLL (the difference in stability is so large that it cannot be compared), while using RML and Lecitase^®^ Ultra differences, even very significant, are not so high (e.g., the stability of the new preparation for Lecitase^®^ is more than fifteen folds lower at pH 5, twenty at pH 7 and less than two fold at pH 9).

This negative effect of the MCI GEL^®^ CHP20P on the thermal stability of the enzymes was also found using CALB [36], and was explained due to their very hydrophobic nature, that may facilitate the stabilization of partially unfolded enzyme structures [23,52].

**Table 3:** Thermal stability of the different biocatalysts at different pH values and 45 °C (given as half-lives in min).

Biocatalyst	pH		
	5	7	9
TLL-OS *	>90% after 10h	>90% after 10h	>90% after 10h
TLL- MCI	23	20	23
RML-OS	1800	750	26
RML-MCI	180	105	30
LU-OS **	510	900	30
LU-MCI **	30	45	24

Experiments were performed as described in the methods section. OS: Octyl-Sepharose^®^; MCI: MCI GEL^®^ CHP20P. * At 45 °C the inactivation rate was too slow to calculate half live for the preparation TLL-OS; ** Temperature was increased to 50 °C to accelerate the inactivation. The results are the mean of three independent experiments and the experimental error was never over 10%.

### 2.4. Stability of MCI GEL^®^ CHP20P and octyl-Sepharose^®^ Lipase Preparations in Presence of acetonitrile

**Figure 2:**
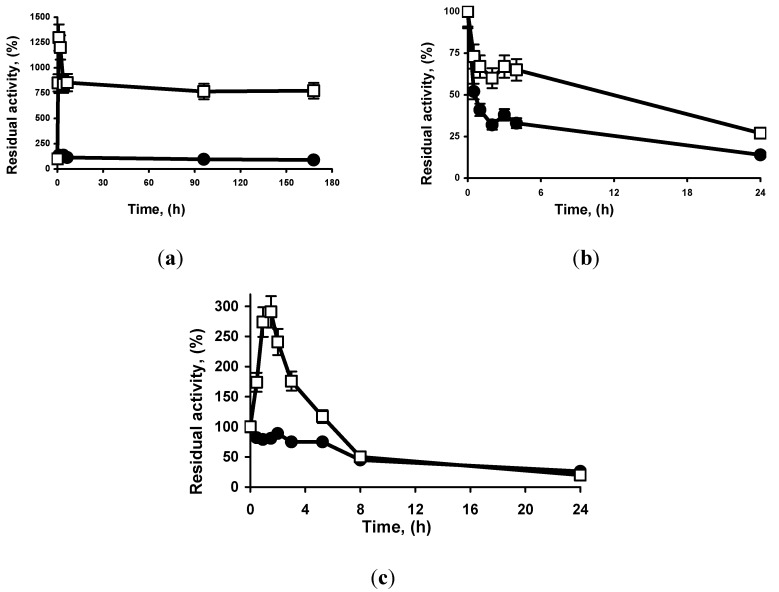
Effect of the incubation in the presence of acetonitrile of different lipase preparations on the enzyme activity. Experiments were carried out as described in methods at 25 °C and pH 7. (**a**): TLL (80% acetonitrile); (**b**): RML (30% acetonitrile); (**c**): Lecitase^®^. (30% acetonitrile) Circles: octyl-Sepharose^®^, Squares: MCI GEL^®^ CHP20P. The results are the mean of 3 independent experiments and the experimental error was never over 5%.

Figure 2 shows the effect of the incubation in the presence of acetonitrile on the activity of the different immobilized enzymes (measured in aqueous medium).

Using TLL, both preparations were very stable on high concentrations of acetonitrile. In fact, the incubation in 80% acetonitrile produced a very significant and progressive initial increase in activity when the enzyme was immobilized on MCI GEL^®^ CHP20P, even by a 12-fold factor after 1–2 h. Using octyl-Sepharose^®^, there was also an increase in activity, but it is much lower: under a 40% after 2 h. After 1 week of incubation at this very high cosolvent concentration, the new preparation presented almost 8 times more activity than the initial one, while the octyl-Sepharose^®^ TLL activity had decreased to 90%.

Using RML, the incubation in 30% acetonitrile was enough to cause a decrease in enzyme activity for both immobilized enzymes, with a slower decrease in enzyme activity using the new preparation (half-lives of 0.5 h for octyl-Sepharose^®^-RML and 10 h for MCI GEL^®^ CHP20P-RML) (Figure 2).

Using Lecitase^®^, the drop in activity was very rapid and similar for both preparations in 50% acetonitrile, while using 30% of solvent MCI GEL^®^ CHP20P-Lecitase^®^ Ultra exhibited an initial increase in activity followed by a progressive decrease in activity (Figure 2). This initial hyperactivation was not observed using octyl-Sepharose^®^. After 6 h, while the octyl-Sepharose^®^ preparation maintained only 50% of the initial activity, the new preparation exhibited more than 100%.

This higher stability in the presence of organic solvent of the lipases immobilized on the styrene divinylbenzene matrix can be explained by the higher strength of the enzyme support-interaction, which cannot be broken by the concentration of the used organic solvents. The initial increase in activity for some of the lipases suggests that the adsorption to the support may be too strong, favoring a partial blocking of the active center, and that the presence of the solvents can produce some relaxing on this adsorption, producing the reported initial increase of activity. However, direct conformational changes of the enzyme structure cannot be discarded [20].

### 2.5. Enzyme Activity Versus other Substrates

As it has been found in many different examples, the immobilization may greatly alter the enzyme specificity, mainly using lipases, even if the mechanism of immobilization is similar [17,19,20]. For this reason, the activities of the enzyme derivatives *versus* three structurally different substrates were evaluated. First, a hydrophobic aliphatic ester, ethyl hexanoate was used. Second, methyl phenylacetate, where the acid is an aromatic cycle, was assayed. And finally, the most complex one, racemic methyl mandelate, that presents an aromatic ring and a hydroxyl group in alpha position regarding the carboxyl group, was utilized as substrate. For TLL and RML; the commercial preparations were used to compare the final activities, even though the home-made preparation presented only 50 mg/g of support of enzyme to reduce the diffusion problems (around 20%–30% of the maximum loading, as shown in Table 1).

Table 4 shows the specific activities of the different lipase preparations under different conditions. Analyzing the results using TLL, the specific activity of the new preparation on the aliphatic ester remained lower than that observed using octyl-Sepharose^®^-TLL at pH 5 (almost 4-fold) or pH 7 (about 7-fold). However, at pH 8.5 the new preparation has a slightly higher specific activity. Using the ester of phenylacetic, the new preparation is around 3 times less active than the octyl-Sepharose® per mg of enzyme. However, using mandelic acid, the preparation becomes the most active, 2.5 fold at pH 5 and 7, and 5.5 at pH 8.5. Even the optimal pH value depends on the substrate and the biocatalyst, and that occurred even though none of the used compounds has ionizable groups in the range of the utilized conditions. The highest activity was found at pH 5 using the aliphatic ester for both biocatalysts, at pH 7 using all other combinations, except methyl mandelate and the new biocatalyst that presented the highest activity at pH 8.5.

Comparing with the commercial preparation (Table 5), Lipozyme^®^ TL-IM, and using ethyl hexanoate as substrate, the mass activity of the new biocatalyst is around 5-6 folds higher than that of the commercial one, depending on the pH value. Using methyl phenyl acetate the differences are very short, while using methyl mandelate, the differences are large: 90-folds at pH 5 and 7 and over 250 at pH 8.5, favoring the new catalyst.

In the case of RML, the immobilization on MCI GEL^®^ CHP20P-RML produced a decrease of the activity *versus* ethyl hexanoate at pH 7 (35-folds), and a little decrease at pH 7 (2-folds) while at pH 8.5 is 50% more active (Table 4). Using methyl phenylacetate the new preparation is 7-fold more active at pH 7, 3.5-fold at pH 5 and the octyl-Sepahrose^®^-RML has no detectable activity at pH 8.5, while using the new biocatalyst the activity was similar to that found at pH 7.

**Table 4:** Specific activity of the different biocatalysts *versus* ethyl hexanoate (EH), methyl phenylacetate (MP) and methyl mandelate (MM) under different conditions.

Biocatalyst/substrate	pH		
	5	7	8.5
TLL-OS/EH	480	165	17
TLL- MCI/EH	125	25	20
TLL-OS/MP	0.12	0.21	0.09
TLL- MCI/MP	0.03	0.06	0.03
TLL-OS/MM	4.8	6	3.1
TLL- MCI/MM	10	16	17.5
RML-OS/EH	671	435	0.13
RML-MCI/EH	333	13	0.2
RML-OS/MP	0.02	0.03	<10^−3^
RML-MCI/MP	0.07	2.1	2.24
RML-OS/MM	4.5	6.2	4.5
RML-MCI/MM	15	105	142
LU-OS/EH	0.04	0.66	0.1
LU-MCI/EH	32	30	29
LU-OS/MP	<0.01	<0.01	<0.001
LU-MCI/MP	4.41	4.5	10
LU-OS/MM	0.55	1.35	11
LU-MCI/MM	5.8	55	47

Experiments were performed as described in the Experimental section. Activity is given in nmol of ester hydrolyzed per minute and per mg of enzyme. OS: Octyl-Sepharose^®^; MCI: MCI GEL^®^ CHP20P.

**Table 5:** Activity per gram of wet biocatalyst of the MCI GEL^®^ CHP20P-lipase preparations (50 mg/g of support) compared to the activity of the respective commercial preparations.

Biocatalyst/Substrate	pH		
	5	7	8.5
IM-TLL/EH	1133	186	150
TLL- MCI/EH	6325	1249	1010
IM-TLL/MP	15	29	9
TLL- MCI/MP	14	31.5	13.5
IM-TLL /MM	6.25	8.5	3.2
TLL- MCI/MM	510	800	870
IM-RML/EH	985	78	17
RML-MCI/EH	16650	642	10.2
IM-RML/MP	7820	2310	7900
RML-MCI/MP	3.7	105	112
IM-RML/MM	75	490	590
RML-MCI/MM	712	5250	7100

Experiments were performed as described in the Exprimental section. Activity is given in nmol of ester hydrolyzed per minute and per wet gram of support. IM-TLL (Lipozyme^®^ TL-IM) and IM-RML (Lipozyme^®^ RM-IM) were products from Novozymes, MCI preparations are those obtained by immobilization on MCI GEL^®^ CHP20P. EH: ethyl hexanoate; MP: methyl phenylacetate; MM: methyl mandelate.

Using the most complex substrate, methyl mandelate, the activity kept a similar trend but the differences are higher in favor of the new preparation; at pH 7 the new preparation is 17 fold more active, at pH 8.5 is over 30 times more active, while at pH 5 differences become reduced (3-fold).

Compared to the commercial preparation (Table 5), Lipozyme^®^ RM-IM, the differences are huge and dependent on the substrate and the pH. Using the aliphatic ester, the new preparation is around 7–8 folds more active at pH 5 and 7, while at pH 8.5 have a 70% more activity. Using methyl phenylacetate, the commercial preparations is much more active than the new preparation: at pH 7 it is more than 20 folds, but at pH 5 it is over 2000 folds and at pH 8.5 more than 70 times. This situation is reversed using methyl mandelate, the MCI GEL^®^ CHP20P-RML is over 8–12 fold more active, depending on the pH value.

In the case of Lecitase^®^ Ultra (Table 4), the immobilization of the enzyme on MCI GEL^®^ CHP20P, the three substrates permitted a much higher activity than the enzyme immobilization on octyl-Sepharose®, in opposition to the results using pNPB. Using ethyl hexanoate, the activity increased by a factor of 45 (at pH 7), 800 (at pH 5) or 3000 (at pH 8.5). Using methyl phenylacetate, the activity could not be determined using octyl-Sepharose^®^ Lecitase^®^, while the activity using MCI GEL^®^ CHP20P-Lecitase^®^ was around 10-fold lower than using the aliphatic ester (at leats difference in activity was a 5000 fold factor). Using methyl mandelate, differences in activity were not so large, at pH 7, the new preparation was 40 fold more active, while at pH 5, it was more than 10 folds more active, and at pH 8.5 it was only 4.5 fold more active.

## 3. Experimental

### 3.1. Materials

The enzymes used in this work were *Thermomyces lanuginosus* lipase free or immobilized in a silicate support (Lipozyme^®^ TL-IM); *Rhizomucor miehei* lipase free or immobilized in an anion-exchange resin (Lipozyme^®^ RM-IM) and free Lecitase^®^ Ultra. All of them were kindly donated by Novozymes (Madrid, Spain). Octyl-Sepharose^®^ crosslinked 4% beads were from GE Healthcare (Uppsala, Sweden). Styrene–divinylbenzene MCI GEL^®^ CHP20P beads *p*-nitrophenyl butyrate (*p*-NPB), methyl mandelate, ethyl hexanoate, and methyl phenylacetate were from Sigma Chemical Co. (St. Louis, MO, USA). All experiments have been performed, at least, by triplicate and the values are the mean values. Standard error was under 10% in all cases.

### 3.2. Standard Determination of Enzyme Activity

This assay was performed by measuring the increase in absorbance at 348 nm (isosbestic point) produced by the released *p*-nitrophenol in the hydrolysis of 0.4 mM *p*-nitrophenyl butyrate (p-NPB) in 100 mM sodium phosphate at pH 7.0 and 25 °C (ε under these conditions is 5,150 mol^−1^·cm^−1^). To start the reaction, 50–100 µL of lipase solution or suspension was added to 2.5 mL of substrate solution. One unit of activity (U) was defined as the amount of enzyme that hydrolyzes 1 µmol of *p*-NPB per minute under the conditions described previously. Protein concentration was determined using Bradford’s method [53] and bovine serum albumin was used as the reference.

In the studies of pH effects on the enzyme activity, the protocol was similar but the buffer in the measurements was changed according to the pH value: sodium acetate at pH 5, sodium phosphate at pH 6–8 and sodium borate at pH 9–10. At 25 °C, all the preparations remained fully active after incubation for several hours at any of these pH values.

### 3.3. Immobilization of Lipases

Styrene-divinylbenzene support is so hydrophobic that water hardly can directly penetrate into their pores. Thus, a sample of 10 g of this support was suspended in 100 mL of acetonitrile for 1 h under mild stirring, and then 100 mL of water were added. After 1 additional hour of mild stirring, the supports were filtered and re-suspended in 100 mL pure water for another hour under stirring. Finally, the supports were washed in a glass funnel five times with 5 volumes of water and stored at 4 °C.

Immobilization was performed using 3 or 50 mg of protein per g of wet support. The commercial samples of the lipases were diluted in the corresponding volume of 5 mM sodium phosphate at pH 7. Then, the supports were added. The activity of both supernatant and suspension was followed using *p*-NPB (see Section 3.2). After immobilization the suspension was filtered and the supported lipase was washed several times with distilled water.

### 3.4. Determination of the Loading Capacity of the Different Supports

The different supports (2 g) were added to a 400 mL solution of the corresponding lipase solution having from 0.1 to 1 mg protein/mL in 5 mM sodium phosphate at pH 7 and 25 °C, adding 0.01% sodium azide to prevent microbial contamination. Samples of supernatant were taken periodically to measure its activity by the *p*-NPB assay and immobilization was considered to be complete when no significant changes in enzyme activity in the supernatant were detected after 4 h, with a maximum of 24 h.

### 3.5. Thermal Inactivation of Different Lipase Immobilized Preparations

To check the stability of enzyme derivatives, immobilized enzyme (1 g) was suspended in 10 mM of sodium acetate (5 mL) at pH 5, sodium phosphate at pH 7 or sodium carbonate at pH 9 at the indicated temperatures. Periodically, samples were withdrawn and the activity was measured using *p-*NPB. Half-lives were calculated from the observed inactivation courses.

### 3.6. Inactivation of Different Enzyme Preparations in the Presence of Acetonitrile

Enzyme preparations were incubated in mixtures of acetonitrile in 100 mM Tris–HCl at pH 7 and 25 °C (pH previously adjusted using NaOH) to proceed with the inactivation. Periodically, samples were withdrawn and the activity was measured using *p*-NPB. The acetonitrile present in the samples had no significant effect during enzyme activity determination.

### 3.7. Hydrolysis of Ethyl Hexanoate

Enzyme activity was determined by using ethyl hexanoate; 200 mg of the immobilized preparations were added to 0.6 mL of 25 mM substrate in 50 mM buffer containing 50% CH_3_CN. The buffer was sodium acetate at pH 5, sodium phosphate at pH 7 and sodium carbonate at pH 8.5. All experiments were carried out at 25 °C under continuous stirring. The conversion degree was analyzed by RP-HPLC (Spectra Physics SP 100 coupled with a Spectra Physics SP 8450 UV detector, (Thermo, Santa Clara, CA, USA) using a Kromasil C18 (15 cm × 0.46 cm) column. Samples (20 μL) were injected and eluted at a flow rate of 1.0 mL/min using acetonitrile /10 mM ammonium acetate aqueous solution (50:50, v/v) and pH 3.2 as mobile phase and UV detection was performed at 208 nm. Hexanoic acid has a retention volume of 3.4 mL while ester has a retention volume of 14.2 mL. One unit of enzyme activity was defined as the amount of enzyme necessary to produce 1 nmol of hexanoic acid per minute under the conditions described above. Activity was determined by triplicate with a maximum conversion of 20%–30%, and data are given as average values.

### 3.8. Hydrolysis of Methyl Phenylacetate

Enzyme activity was determined by using methyl phenylacetate; 200 mg of the immobilized preparations were added to 0.6 mL of 5 mM substrate in 50 mM buffer containing 50% CH_3_CN. The buffer was sodium acetate at pH 5, sodium phosphate at pH 7 and sodium carbonate at pH 8.5. All experiments were carried out at 25 °C under continuous stirring. The conversion degree was analyzed by RP-HPLC (Spectra Physics SP 100 coupled with a Spectra Physics SP 8450 UV detector) using a Kromasil C18 (15 cm × 0.46 cm) column. Samples (20 μL) were injected and eluted at a flow rate of 1.0 mL/min using acetonitrile, 10 mM ammonium acetate aqueous solution (35:65, v/v) and pH 2.8, as mobile phase and UV detection was performed at 230 nm. Phenylacetic acid has a retention volume of 4.2 mL while the ester has a retention volume of 12.5 mL. One unit of enzyme activity was defined as the amount of enzyme necessary to produce 1 nmol of phenylacetic acid per minute under the conditions described above. Activity was determined by triplicate with a maximum conversion of 20%–30%, and data are given as average values.

### 3.9. Hydrolysis of Methyl Mandelate

Enzyme activity was also determined by using methyl mandelate. The immobilized preparations (200 mg) were added to 50 mM substrate in 50 mM sodium acetate (1 mL) at pH 5, 50 mM sodium phosphate at pH 7 or 50 mM sodium carbonate at pH 8.5 and 25 °C under continuous stirring. The conversion degree was analyzed by RP-HPLC (Spectra Physics SP 100 coupled with a Spectra Physics SP 8450UV detector) using a Kromasil C18 (15 cm × 0.46 cm) column. Samples (20 μL) were injected and eluted at a flow rate of 1.0 mL/min using acetonitrile/ 10 mM ammonium acetate (35:65, v/v) at pH 2.8 as mobile phase and UV detection was performed at 230 nm. The acid has a retention volume of 2.4 mL while the ester has a retention volume of 4.2 mL One unit of enzyme activity was defined as the amount of enzyme necessary to produce 1 nmol of mandelic acid per minute under the conditions described above. Activity was determined by triplicate with a maximum conversion of 20%–30%, and data are given as average values.

## 4. Conclusions

MCI GEL^®^ CHP20P beads are a support that permits very high lipase (or Lecitase^®^ Ultra) loading and a very rapid immobilization. The hydrophobic nature of this matrix produces that the thermal stability is lower than that obtained immobilizing the enzymes on the more hydrophilic octyl-Sepharose^®^, while the stability in the presence of organic cosolvents is greatly improved using the support under evaluation.

Regarding the effects on activity, the results are not so clear. Using the same enzyme, for some substrates the activity becomes much higher using MCI GEL^®^ CHP20P than that obtained using octyl-Sepharose®, while using other substrates the situation is the opposite. This difference in activity is of several orders of magnitude, and so large differences in the substrate specificity has not been reported to date, and that has been obtained just changing the support, but maintaining the interfacial activation of the enzyme on the support as the reason for enzyme immobilization. That means that the only reason for this drastic changes, found for these three enzymes and previously described using CALB, are based in a different conformation of the immobilized enzyme, whose open form will be stabilized on a different structure. In fact, each enzyme/support composite has complete different properties, including response to change in reaction variables (e.g., changes in pH value), behave as almost fully different biocatalysts.

These changes show the potential of immobilization to tune lipase properties, and the great potential of this new support to immobilize lipases for many applications. Coupling these results to the high hydrophobicity of the support, that should reduce the undesired adsorption of water, glycerin, etc, that are a problem in certain processes [54], this matrix may have indubitable interest as a matrix to immobilize lipases for many different processes. However, the effects need to be experimentally determined, as the current technology did not permit to foresee the better matrix to immobilized a lipase for a determined process [20].
